# Overexpression of *GmWRKY172* enhances cadmium tolerance in plants and reduces cadmium accumulation in soybean seeds

**DOI:** 10.3389/fpls.2023.1133892

**Published:** 2023-03-09

**Authors:** Peiqi Xian, Yuan Yang, Chuwen Xiong, Zhibin Guo, Intikhab Alam, Zihang He, Yakun Zhang, Zhandong Cai, Hai Nian

**Affiliations:** ^1^The State Key Laboratory for Conservation and Utilization of Subtropical Agro-Bioresources, South China Agricultural University, Guangzhou, China; ^2^Guangdong Laboratory for Lingnan Modern Agriculture, Guangzhou, China; ^3^The Key Laboratory of Plant Molecular Breeding of Guangdong Province, College of Agriculture, South China Agricultural University, Guangzhou, China; ^4^Department of Grassland Science, College of Forestry and Landscape Architecture, South China Agricultural University, Guangzhou, China; ^5^Hainan Yazhou Bay Seed Lab, Hainan, China

**Keywords:** WRKY transcription factor, heavy metal stress, Cd tolerance, Cd translocation, RNA-sequencing, soybean

## Abstract

**Introduction:**

Cadmium (Cd) stress is a significant threat to soybean production, and enhancing Cd tolerance in soybean is the focus of this study. The WRKY transcription factor family is associated with abiotic stress response processes. In this study, we aimed to identify a Cd-responsive WRKY transcription factor *GmWRKY172* from soybean and investigate its potential for enhancing Cd tolerance in soybean.

**Methods:**

The characterization of *GmWRKY172* involved analyzing its expression pattern, subcellular localization, and transcriptional activity. To assess the impact of *GmWRKY172*, transgenic Arabidopsis and soybean plants were generated and examined for their tolerance to Cd and Cd content in shoots. Additionally, transgenic soybean plants were evaluated for Cd translocation and various physiological stress indicators. RNA sequencing was performed to identify the potential biological pathways regulated by GmWRKY172.

**Results:**

*GmWRKY172* was significantly upregulated by Cd stress, highly expressed in leaves and flowers, and localized to the nucleus with transcriptional activity. Transgenic plants overexpressing *GmWRKY172* showed enhanced Cd tolerance and reduced Cd content in shoots compared to WT. Lower Cd translocation from roots to shoots and seeds was also observed in transgenic soybean. Under Cd stress, transgenic soybean accumulated less malondialdehyde (MDA) and hydrogen peroxide (H_2_O_2_) than WT plants, with higher flavonoid and lignin contents, and peroxidase (POD) activity. RNA sequencing analysis revealed that many stress-related pathways were regulated by GmWRKY172 in transgenic soybean, including flavonoid biosynthesis, cell wall synthesis, and peroxidase activity.

**Discussion:**

Our findings demonstrated that GmWRKY172 enhances Cd tolerance and reduces seed Cd accumulation in soybean by regulating multiple stress-related pathways, and could be a promising candidate for breeding Cd-tolerant and low Cd soybean varieties.

## 1 Introduction

As one of the most economically important crops, soybean (*Glycine max* (L.) Merr.) has been a key source of high-quality protein and other nutrients for both humans and livestock around the world. Nevertheless, soybean is susceptible to the effects of cadmium (Cd) stress and readily stores Cd in its seeds (Arao et al., 2003; Wang et al., 2014). Cd is a nonessential element for plants and has become one of the most hazardous heavy metals in the environment due to rapid industrial activity, wastewater irrigation, atmospheric deposition, and other human activities (Zhang et al., 2020b; Zhang et al., 2021; Zulfiqar et al., 2022). According to a nationwide survey on the status of soil contamination in China, approximately 7% of soil samples exceed the cadmium standard, which ranks first among all types of heavy metals and metalloids (Zhao et al., 2015). Soils with elevated Cd concentrations hamper plant growth and development, resulting in a reduction in crop yield. Additionally, Cd can easily be absorbed by crops and be transmitted to humans through the food chain, thereby potentially posing a threat to human health (Shahid et al., 2017). In the last century, the World Health Organization (WHO) recommended limiting Cd intake to less than 7.0 µg/kg body weight/week (WHO, 1993). Therefore, it is necessary to decipher the mechanisms of plant Cd tolerance/accumulation, and accelerate the pace of genetic improvement of Cd tolerance.

Plants have developed elaborate regulatory mechanisms to perceive Cd stress signals and adjust Cd tolerance-related pathways. These mechanisms mainly include enhancing antioxidant activity, regionalization, tissue immobilization, metallothionein formation, and active efflux (Wu et al., 2016b; Rizwan et al., 2017; Shahid et al., 2017). In recent decades, the response of plants to Cd stress has been intensively studied and it has been shown that the proteins in plant response to Cd stress were mainly related to energy metabolism, antioxidant, transport, signal transduction, and transcriptional regulation (Xu et al., 2017; Salas-Moreno et al., 2022). Exposure of plants to Cd stress leads to the overproduction of reactive oxygen species (ROS), impairing the redox homeostasis of cells. By producing antioxidant enzymes and non-enzymatic antioxidants like peroxidase, superoxide dismutase, glutathione, flavonoids, etc., the strong antioxidant defense systems in plants are able to tightly regulate the quantity of ROS and protect themselves from oxidative damage (Zhu et al., 2020). It has been reported that ROS are also significant signaling molecules that interact with other signaling components such as plant hormones and calcium ions to coordinate complex signaling cascades in plants (Xia et al., 2015; Sewelam et al., 2019). In addition, the absorption of Cd in roots and its transportation within plants require a number of heavy metal transporters. ATP-binding cassette transporters (ABCs) and some ion channel-related proteins, like the iron-regulated transporter family (IRT), the Zn-Fe transporter family (ZIP), and the natural resistance-associated macrophage protein family (NRAMP) have been identified as Cd transporters (Kim et al., 2007; Sasaki et al., 2012; Wu et al., 2016a; Fu et al., 2019; Zhang et al., 2020b). At the transcriptional level, transcription factors (TFs) are essential components in the regulatory network of Cd detoxification and Cd tolerance (DalCorso et al., 2010).

TFs are regulatory proteins that function to activate or suppress the expression of downstream genes by binding to the promoter regions of target genes (Li et al., 2020). They are involved in various physiological processes of plants, such as growth, development, and signal transduction triggered by stress environments (Millard et al., 2019). Currently, a growing number of TFs, including members of the myeloblastosis protein (MYB), basic leucine Zipper (bZIP), ethylene-responsive factor (ERF), heat shock transcription factor (Hsf), WRKY, and other families, have been reported to implicate in regulating plant responses to Cd stress (Lin et al., 2017; Sheng et al., 2019; Zhang et al., 2019; Chen et al., 2020; Lu et al., 2022). WRKY is one of the largest TF families involved in biotic and abiotic stress responses in plants, defined by a highly conserved sequence WRKYGQK at the N-terminus and a zinc-finger motif at the C-terminus (Rushton et al., 2010; Khoso et al., 2022). According to earlier research, WRKY TFs can interact with target genes involved in stress regulation to impact plant stress tolerance. The promoter regions of these target genes harbor the W-box core sequence TTGACC/T, which is the specific recognition site of WRKYs (Phukan et al., 2016; Jiang et al., 2017). For example, PbrWRKY53 from *Pyrus betulaefolia* positively regulated the expression of *PbrNCED1* in conjunction with the W-box motifs of the promoter to enhance the production of vitamin C, which may contribute to the improvement of plant drought tolerance (Liu et al., 2019). In *Arabidopsis thaliana*, WRKY12 binds directly to the W-box of the *GSH1* promoter, repressing the expression of genes associated with glutathione-dependent phytochelatin production indirectly for the negative regulation of Cd tolerance (Han et al., 2019). WRKY13 was proved to directly regulate Cd tolerance in *Arabidopsis* by targeting PDR8 (Sheng et al., 2019). Besides, it has been suggested that the combination of ThVHAc1 and its upstream regulator ThWRKY7 could improve the tolerance of Cd stress in woody plants (Yang et al., 2016). Furthermore, the WRKY gene family also functions as an efficient co-regulatory network. As a group, WRKY genes may share common signal transduction pathways and link together to form a transcriptional network with both positive and negative feedback and feed-forward loops (Eulgem and Somssich, 2007; Berri et al., 2009). Even though many studies have shown that WRKY TFs play a role in the response of plants to abiotic stress, not enough research has been done on the function and molecular mechanism of soybean WRKY TFs in Cd stress response.

Previously, we performed a transcriptome analysis on Cd-treated soybean and identified 29 WRKY genes, of which *Glyma.18g213200* displayed substantial up-regulation during Cd treatment (Cai et al., 2020). However, the function of this gene, named *GmWRKY172*, is unclear. In this work, we overexpressed it in *Arabidopsis thaliana* and soybean, and investigated its role in Cd tolerance. Overexpression of *GmWRKY172* did not alter the growth characteristics under normal growth condition, however, it significantly decreased the toxic effect of Cd in *Arabidopsis thaliana* and soybean upon Cd stress. Phenotypic, physiological, and transcriptome analysis showed that overexpression of *GmWRKY172* improved the antioxidant capacity of soybean, while reduced ROS accumulation and enhanced the capacity of the root cell wall to bind Cd. Altogether, our findings characterized the function of *GmWRKY172* in response to Cd stress, and indicated that GmWRKY172 improves Cd tolerance by activating the signaling network of the antioxidant system and reduces Cd accumulation in shoots and seeds by fixing Cd in the root cell wall, providing a crucial directive for the other researches of WRKYs and the breeding of Cd-tolerant and low Cd soybean.

## 2 Materials and methods

### 2.1 Vector construction and plant genetic transformation

The cDNA sequences of *GmWRKY172* (*Glyma.18G213200*) were isolated using specific primers and inserted into the *Xba* I and *Sac* I sites of the pTF101.1 vector using the ClonExpress^®^ II One Step Cloning Kit (C112, Vazyme, Nanjing, China). The resulting plasmid was mobilized into *Agrobacterium* strains GV3101 and EHA101 by heat shock and subsequently used to transform *Arabidopsis thalian* and soybean (Clough and Bent, 1998; Li et al., 2017a). The complete coding sequence of *GmWRKY172* was cloned into the vectors of pCAMBIA 1302, pGADT7, and pGreenII 62-SK using the same way for the determination of subcellular localization, yeast one-hybrid assay, and dual LUC assay, respectively. The promoter fragments containing the W-box or mutated W-box element were amplified by PCR and cloned into the pAbAi vector for yeast one-hybrid assays. The primers used in vector construction are listed in **Table S1**.

### 2.2 Plant materials and treatments

The seeds of soybean variety ZH24 and three T4 generation homozygous transgenic lines (OX4-7, OX6-2, and OX9-5) were surface sterilized with 5% (v/v) sodium hypochlorite and then thoroughly washed before being planted in the soil containing 0.40 mg kg^-1^ Cd or the control soil for short- and long-term experiments in a greenhouse (14-h light/10-h dark photoperiod, average temperature 25-30°C, relative humidity 60-80%). The soil media were collected from the Wengyuan county of Shaoguan (N24°46’, E113°49’, Cd-polluted area) and the farm of South China Agricultural University (N23°15’, E113°34’, control group). Physicochemical properties of soil were determined and presented in **Table S2**. The plant samples were also used for the subsequent RNA-seq and physiological index determination experiments. For the dose-dependent expression pattern analysis, five-day-old soybean seedlings were subjected to a modified 1/2 Hoagland solution (Cai et al., 2020) containing 0, 5, 10, 15, 25, or 50 µM of CdCl_2_ for 4 h.

Surface-sterilized *Arabidopsis thaliana* seeds of ecotype Columbia-0 (WT) and three T3 generation transgenic lines (OX-B6, OX-E2, and OX-H1) were planted in sterilized matrix soil (Jiffy, Oslo, Norway) after three days of vernalization, then grew in an illuminated growth incubator at a temperature of 25°C and a relative humidity of 80%. Three-week-old plants were given the additional treatment of 1/10 Hoagland solution containing 0 or 500 µM CdCl_2_ every three days with 20 mL each. After three times of treatment, the plants were allowed to grow for another 15 days, after which the plant’s fresh weight and Cd concentration were assessed.

### 2.3 Subcellular localization

The Green Fluorescent Protein (GFP) was fused to GmWRKY172, and the resultant construct 35S:GmWRKY172-GFP was transiently expressed in *Nicotiana benthamiana* leaves *via Agrobacterium*-mediated transformation. The nuclear dye (DAPI) and GFP fluorescence signals were detected using laser confocal microscopy (LSM780, Zeiss, Jena, Germany).

### 2.4 Yeast one-hybrid assay

The MATCHMAKER^®^ Gold Yeast One-Hybrid Library Screening System (Clontech) and the YEASTMAKER™ Yeast Transformation System 2 (Clontech) were jointly applied to examine the physical interactions between the promoters and the transcription factors. The bait vector and the pGADT7 prey vector were introduced into Y1H Gold yeast according to the instructions of the manufacturer. The cells were grown in SD/-Leu liquid medium to an OD_600_ of 0.1 before being diluted 10-fold with saline. In order to determine the strength of the interaction, 7 µL of each dilution was spotted on SD/-Leu media plates containing either 0 or 150 ng/mL AbA. Plates were incubated for 3-4 days at 30°C.

### 2.5 Determination of Cd and chlorophyll content

The plant tissue was harvested and dried for 3 h at 105°C and then for 3 days at 80°C. The dry weights of the samples were measured, then the samples were completely digested with extra pure grade HNO_3_/HClO_4_ (87/13, v/v). The ICP-AES 9800 was used to measure the levels of Cd (ICP-AES, inductively coupled plasma-atomic emission spectrometry). Fresh leaf chlorophyll content was measured by Chlorophyll Meter (SPAD-502, Konica Minolta, Japan) and expressed in SPAD units.

### 2.6 Determination of POD activity

The previously described method for extracting antioxidant enzymes from fresh soybean sample was followed (Vaculík et al., 2021). First, powders of the samples were mixed with sodium phosphate buffer (1 mM EDTA), filtered, and centrifuged to obtain the supernatant. The centrifuged supernatant was purified by passing it through a Bio-Rad 7322010 Econo-Pac 10DG Desalting Column (Beijing Noblad Technology Co., LTD). The resulting purified solution was used to measure the activity of POD using the Peroxidase Activity Assay Kit (Sigma-Aldrich, Shanghai, China).

### 2.7 Determination of MDA and H_2_O_2_

The MDA and H_2_O_2_ extraction process followed a previously described protocol (Huang et al., 2021). To obtain the supernatant for MDA and H_2_O_2_ content analysis, each fresh sample was treated with trichloroacetic acid solution and then centrifuged at 4°C. The MDA and H_2_O_2_ contents were determined using the Lipid Peroxidation MDA Assay Kit (Beyotime, Shanghai, China) and Hydrogen Peroxide Assay Kit (Beyotime, Shanghai, China), respectively, according to the manufacturer’s instructions.

### 2.8 Determination of lignin content

The instructions of the kit were followed to determine the lignin content of the sample (Comin Biotechnology, Suzhou, China). The sample was dried at 80°C, then crushed and approximately 2 mg was weighed out into a glass tube. Next, 500 µL bromoacetyl-glacial acetic acid and 20 µL perchloric acid were added to the tube. The tube was then sealed with sealing film, mixed well, and placed in a water bath at 80°C for 40 minutes, with periodic shaking every 10 minutes. After this, the tube was allowed to cool naturally. Add 500 µL NaOH-acetic acid solution to the tube and mixed thoroughly. Then, 20 µL supernatant was taken and mixed with 980 µL glacial acetic acid. Finally, the mixed sample was placed in a colorimetric dish and the absorbance value at 280 nm was determined.

### 2.9 Determination of flavonoid content

The flavonoids were extracted for spectrophotometric analysis using a plant flavonoid kit in accordance with the manufacturer’s instructions (Comin Biotechnology, Suzhou, China). The plant sample was crushed and dried to a constant weight. Next, approximately 0.02 g of the sample was mixed with 2 mL of 60% ethanol and incubated at 60°C with oscillation for 2 hours. After centrifugation at 10,000 × g for 10 minutes at 25°C, the supernatant was collected and allowed to incubate at 25°C for 15 minutes. The absorption value at 510 nm was then determined.

### 2.10 RNA-sequencing and bioinformatics analysis

The soybean seedlings of ZH24 and OX4-7 were sampled to conduct the transcriptomic analysis in LC-Bio company (Hangzhou, China). Total RNA was isolated from the samples using the Spectrum Plant Total RNA Kit (Sigma–Aldrich, St. Louis, MO, United States, STRN10-1KT) and then mixed with fragmentation buffer, which enriched and fragmented mRNA to form first-strand cDNA by further using random hexamer primers. T4 polynucleotide kinase, T4 DNA polymerase, and DNA polymerase I Klenow fragment were then used to repair the ends of the double-stranded cDNAs in preparation for ligating the fragments to attachments with T4 DNA ligase. Following PCR amplification to select and enrich the available fragments for library construction, the fragments were sequenced using an Illumina NovaSeq™ 6000 after qualification and quantification with an Agilent 2100 Bioanalyzer and an ABI StepOnePlus Real-Time PCR System. SOAPaligner/SOAP2 (Li et al., 2009) was utilized to map the clean reads derived from raw reads to soybean reference sequences obtained from the NCBI website (ftp://ftp.ncbi.nlm.nih.gov/genomes/all/GCF/000/004/515/GCF_000004515.5_Glycine_max_v2.1). According to previous methods (Mortazavi et al., 2008), the gene expression level was defined as reads per kilobase per million reads. The gene functions were annotated using the software Blast2GO, which served as a search engine for GO terms (http://www.geneontology.org). In addition, the biological pathways in which the DEGs participated were investigated using the KEGG database (http://www.kegg.jp/kegg).

### 2.11 qRT–PCR assay

Total RNA was isolated from collected samples by applying the FastPure^®^ Cell/Tissue Total RNA Isolation Kit V2 (RC112 Vazyme, Nanjing, China). The quantity of RNA was measured with a NanoDrop 2000 spectrophotometer (Thermo Fisher Scientific, West Palm Beach, FL) before reverse transcription to first-strand cDNA with a PrimeScriptTM RT reagent Kit (RR047, Takara Bio, Shiga, Japan). Next, quantitative real-time PCR (qRT-PCR) assay was performed on CFX96 Real-Time PCR Detection System (Bio–Rad, Hercules, CA, United States) using TB GreenTM Premix Ex TaqTM II (RR820, Takara Bio). The relative gene expression levels were normalized to the reference gene *GmACT3* (GenBank: AK285830.1) or *AtActin2* (At3g18780), and evaluated through the 2^–ΔΔCt^ algorithm (Livak and Schmittgen, 2001). The corresponding primers used in the present study are listed in **Table S1**.

### 2.12 Statistical analysis

Statistical analysis was performed using the analysis of variance on SPSS (version 21), GraphPad Prism^®^ 5 (Version 5.01, GraphPad Software, Inc., USA) was used for calculating the mean and standard deviation of the data. MEGA6.0 software was used to construct the multiple sequence alignment and generate the phylogenetic tree and the results were edited with the GENEDOC software.

## 3 Results

### 3.1 Identification, expression, and molecular characterization of *GmWRKY172*

**Figure 1:**
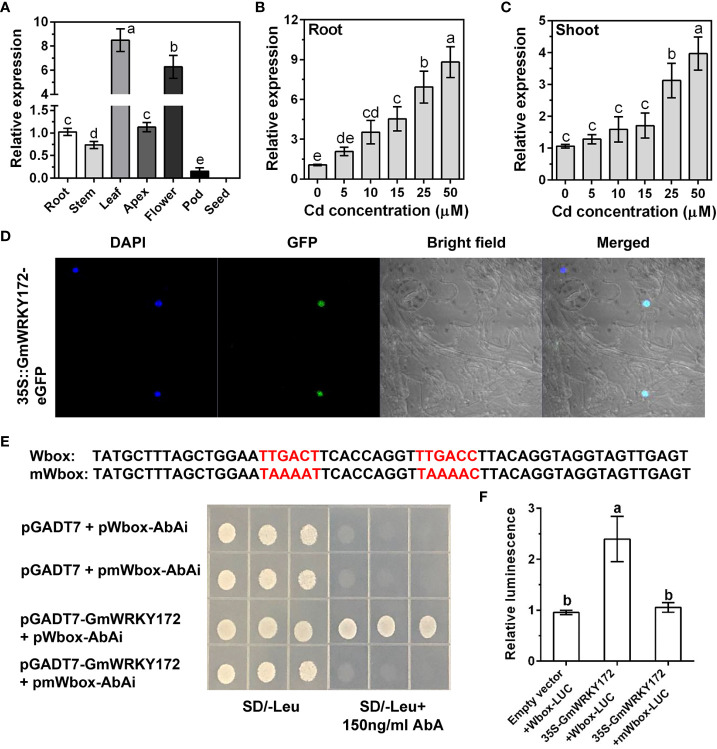
Expression patterns, subcellular localization, and transcriptional activity assays of GmWRKY172. **(A)** Expression analysis of *GmWRKY172* in different tissues of soybean by quantitative reverse transcriptase PCR (qRT-PCR). Dose-dependent expression of *GmWRKY172* in roots **(B)** and leaves **(C)**. Samples were exposed to different Cd concentrations (0, 5, 10, 15, 25 and 50 µM) for 4 hours. **(D)** Subcellular localization of GmWRKY172 in *Nicotiana benthamiana* epidermal cells. **(E)** Yeast one-hybrid analysis for interaction between GmWRKY172 and W-box motifs. The Y1HGold yeast strain was co-transformed with the prey (pGADT7 or pGADT7-GmWRKY172) and the bait (pAbAi-Wbox or pAbAi-mWbox). SD medium lacking leucine with 150 ng/mL AbA was used to determine the interaction between the bait and prey proteins. **(F)** Transcription activity assay in *Nicotiana benthamiana* to examine the interaction between GmWRKY172 and W-box motifs. The relative luminescence was determined by normalizing firefly luciferase activity with Renilla luciferase activity. Data are expressed as mean ± SD (*n* = 3). Different letters indicate statistical significance determined by one-way analysis of variance and Duncan’s test (*P* ≤ 0.05).

We previously obtained a remarkable Cd-induced WRKY gene (*Glyma.18g213200*) named *GmWRKY172* from the soybean transcriptome (Cai et al., 2020). The coding sequence (CDS) of *GmWRKY172* is 900 bp in length and encodes a putative protein of 299 amino acids. Phylogenetic analysis showed that the GmWRKY172 is most closely related to GsWRKY70 (99.0% amino acid similarity). In the model plants *Arabidopsis* and rice, AtWRKY70 and OsWRKY19 have the highest amino acid similarity to GmWRKY172, with 62.9% and 66.3%, respectively (**Figure S1A**). Additionally, GmWRKY172 contains a conserved WRKYGQK domain and a C2-HC domain, as with other three homologs (**Figure S1B**). Tissue level expression analysis showed that *GmWRKY172* was expressed in all examined tissues except for seeds, and the high expression was detected in leaves and flowers (**Figure 1A**). To explore whether *GmWRKY172* is induced by Cd stress, five-day-old soybean seedlings were exposed to varying Cd concentrations for 4 hours. The transcript levels of *GmWRKY172* in both roots and leaves were progressively enhanced with increasing Cd concentration, especially in roots (**Figures 1B, C**), suggesting that *GmWRKY172* is highly responsive of Cd stress.

GmWRKY172 is a putative transcription factor that functions within the nucleus. We next examined the subcellular location of GmWRKY172 by examining the localization of the signal of GmWRKY172-eGFP fusion protein in *Nicotiana benthamiana*. The fluorescence signal of the fusion protein was completely overlapped with the 4’,6-diamidino-2-phenylindole (DAPI) nuclear staining signal, indicating that GmWRKY172 was localized in the nucleus. (**Figure 1D**). As the W-box TTGACT/C was recognized to be the typical binding site for the WRKY gene family, we utilized the yeast one-hybrid (Y1H) test to determine whether there is an interaction between the GmWRKY172 protein and W-box motifs. The result showed that GmWRKY172 was able to interact with W-box (**Figure 1E**). However, when the mutations were introduced into W-box, the interaction disappeared (**Figure 1E**). These results confirmed that GmWRKY172 interacts with W-box. To determine whether GmWRKY172 possesses transcription activation activity, we performed transient expression assays in *Nicotiana benthamiana* using the dual-luciferase reporter system. GmWRKY172 driven by the CaMV35S promoter was used as an effector and a fragment containing W-box motifs or mutated W-box motifs were fused upstream of luciferase (LUC) to form a reporter. As expected, the co-expression of effecter-GmWRKY172 with reporter-Wbox led to a higher LUC activity than that observed in the control and mutated reporter (**Figure 1F**), implying that GmWRKY172 may function as a transcriptional activator.

### 3.2 Overexpression of *GmWRKY172* enhances Cd tolerance in *Arabidopsis thaliana*

**Figure 2:**
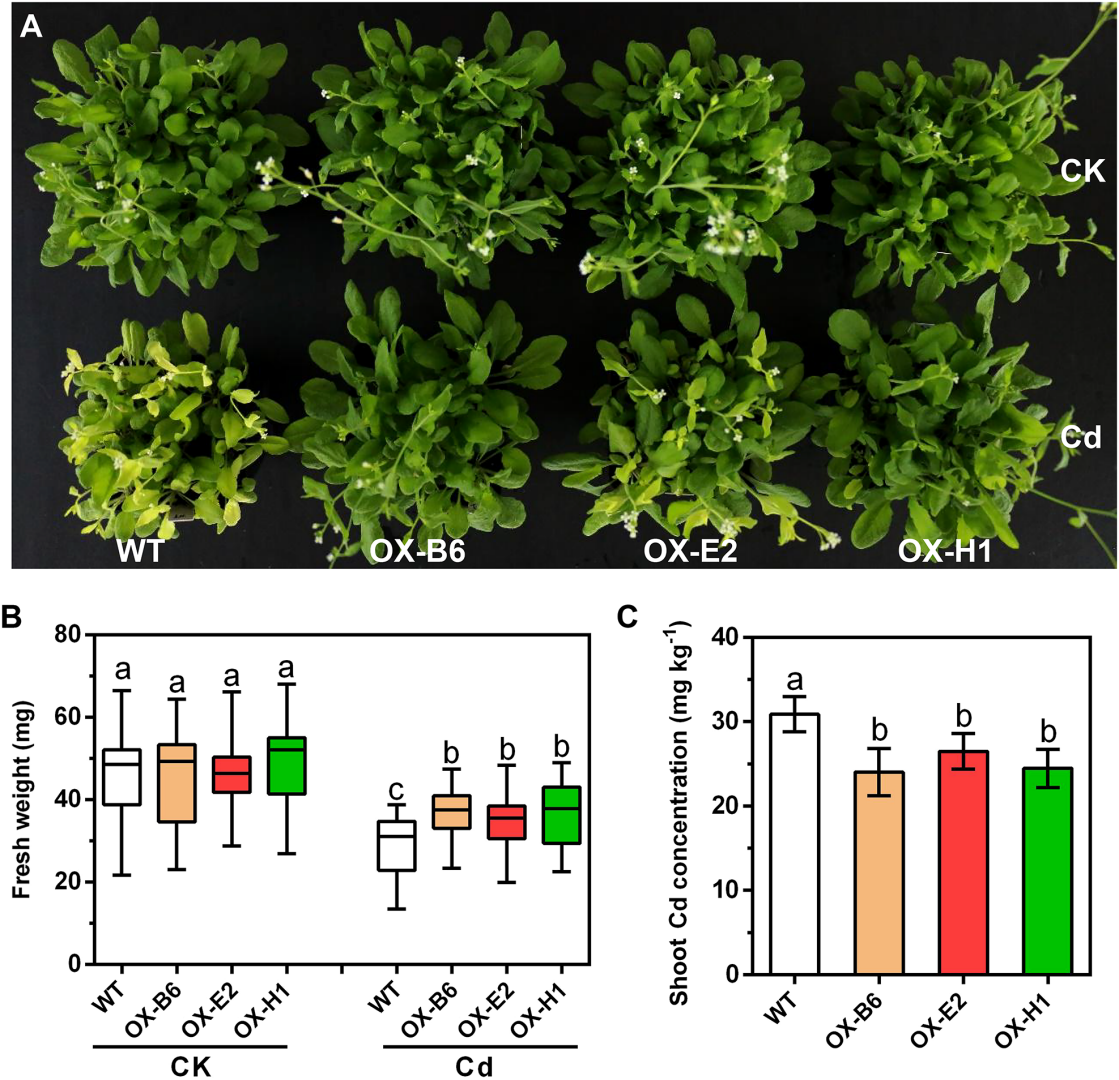
Overexpression of *GmWRKY172* conferred Cd tolerance in *Arabidopsis thaliana*. **(A)** Phenotypes of *Arabidopsis thaliana* transgenic lines (OX-B6, OX-E2, and OX-H1) and WT under normal or Cd-treated conditions. OX refers to *GmWRKY172* overexpression. **(B)** Boxplots for the shoot fresh weight of each plant in different treatment conditions (*n* = 30). **(C)** Shoot Cd concentrations of WT and transgenic lines, data are expressed as mean ± SD (*n* = 5). Different letters indicate statistical significance determined by one-way analysis of variance and Duncan’s test (*P* ≤ 0.05).

To functionally characterize the role of *GmWRKY172* in Cd tolerance, we initially overexpressed it in *Arabidopsis thaliana* Col-0 (WT). Three transgenic lines (OX-B6, OX-E2, and OX-H1) with high GmWRKY172 expression were selected for phenotype analysis in response to Cd stress (**Figure S2**). In the absence of Cd, there were no significant differences in the appearance or biomass of the WT and overexpressing lines. When exposed to Cd, however, overexpressing lines displayed improved Cd tolerance than WT plants (**Figures 2A, B**). Leaf chlorosis and growth inhibition are two typical symptoms of Cd stress in plants (DalCorso et al., 2008; Gutsch et al., 2020). Under Cd stress, the chlorosis level of WT was greater than that of overexpressing lines, and the fresh weight of WT was less than that of overexpressing lines. Consistently, Cd contents in the plant shoots of the three transgenic lines were 14.26% to 22.23% lower than those of the WT (**Figure 2C**). These results indicate that overexpression of *GmWRKY172* in *Arabidopsis thaliana* enhances Cd tolerance and decreases Cd concentration in the plant shoots, which drove us to conduct in-depth research in soybeans.

### 3.3 Overexpression of *GmWRKY172* reduces Cd translocation in soybean

**Figure 3:**
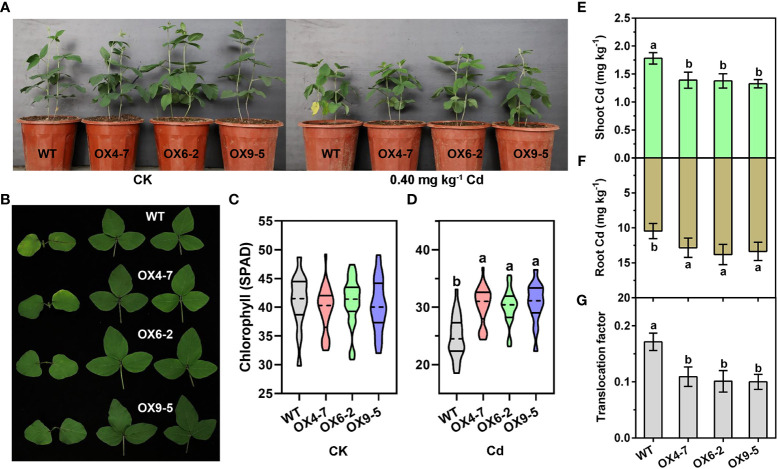
Overexpression of *GmWRKY172* reduced Cd accumulation in the shoots of soybean. **(A, B)** Phenotypes of soybean transgenic lines (OX4-7, OX6-2, OX9-5) and WT under normal or Cd-treated conditions. OX refers to *GmWRKY172* overexpression. The chlorophyll contents of each plant were measured under normal **(C)** or Cd-treated **(D)** conditions (*n* = 30). Cd contents in the shoot **(E)** and root **(F)** of WT and transgenic lines were measured after 25 days of Cd treatment. **(G)** The root-to-shoot translocation factor of Cd (ratios of shoot/root Cd contents) in the WT and transgenic lines. Data are expressed as mean ± SD, *n* = 3 **(E–G)**. Different letters indicate statistical significance determined by one-way analysis of variance and Duncan’s test (*P* ≤ 0.05).

Since overexpression of *GmWRKY172* in *Arabidopsis thaliana* reduced Cd accumulation in shoots, we thus hypothesized that GmWRKY172 might be involved in regulating Cd translocation from roots to shoots/seeds. To test this hypothesis, we generated three stable transgenic soybean lines overexpressing *GmWRKY172*, namely OX4-7, OX6-2, and OX9-5. Analysis showed that GmWRKY172 transcripts were up-regulated in transgenic soybean lines by an average of 2.85-fold (**Figure S3**). It was showed that the vegetative growth of both the WT and transgenic lines were significantly inhibited by a short-term Cd treatment (25 days) (**Figure 3A**). However, *GmWRKY172* overexpressing lines (*GmWRKY172*-OX) had considerably less chlorosis than WT plants, particularly in the first true leaf (**Figure 3B**). Exposure to Cd caused a loss in chlorophyll (SPAD values) of 39.48% in the WT leaves, yet only 23.51-26.47% in the transgenic lines (**Figures 3C, D**). To explore the distribution and translocation of Cd in plants, ICP-AES 9800 was used to measure the Cd content of roots and shoots. The three transgenic lines have higher root Cd levels than WT, but lower shoot Cd content. Furthermore, the Cd translocation factor from roots to shoots was significantly lower in transgenic lines (0.10-0.12) compared to WT (0.17) (**Figures 3E–G**).

**Figure 4:**
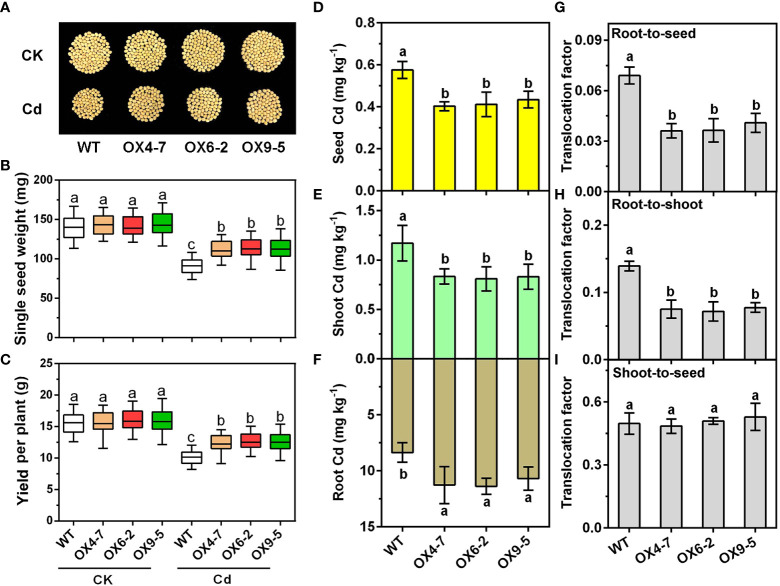
Overexpression of *GmWRKY172* reduced Cd accumulation in seeds of the soybean. Phenotypes of mature seeds **(A)**, single seed weight **(B)**, and yield per plant **(C)** of WT and *GmWRKY172*-OX lines under normal or Cd-treated conditions (*n* = 20). Cd contents in seed **(D)**, shoot **(E)**, and root **(F)** of WT and transgenic lines were measured after 90 days of Cd treatment. The root-to-seed **(G)**, root-to-shoot **(H)**, and shoot-to-seed **(I)** translocation factor of Cd in the WT and transgenic lines. Data are expressed as mean ± SD, *n* = 3 **(D–I)**. Different letters indicate statistical significance determined by one-way analysis of variance and Duncan’s test (*P* ≤ 0.05).

Long-term Cd treatment tests (90 days) were also conducted to explore the effect of GmWRKY172 on soybean yield and grain Cd contents in response to Cd stress. In the control group, there was no significant difference between the WT and transgenic lines in terms of yield per plant and single seed weight. However, the transgenic lines were significantly superior to the WT in the soil containing 0.40 mg kg^-1^. The three transgenic lines increased single seed weight by 22.72%, 25.13%, and 24.99%, respectively, and yield per plant by 22.17%, 25.40%, and 25.54%. (**Figures 4A–C**). Additionally, we also analyzed the concentrations of Cd in roots, shoots, and seeds. The transgenic lines OX4-7, OX6-2, and OX9-5 displayed greater root Cd concentrations than the WT by 34.72%, 36.07%, and 27.77%, respectively. In contrast, Cd accumulation in shoots fell by 28.68%, 30.87%, and 29.05%, and in soybean seeds by 30.00%, 28.55%, and 24.56% (**Figures 4D–F**). The translocation factor showed that Cd translocation from roots to shoots and from roots to seeds was decreased by 44.40-48.65% and 40.81-47.67% respectively, and no significant changes were found from shoots to seeds (**Figures 4G–I**). Taken together, these findings suggest that overexpression of *GmWRKY172* limits Cd translocation in plants, probably by retaining Cd in plant roots.

### 3.4 Overexpression of *GmWRKY172* leads to extensive transcriptional reprogramming of stress-responsive genes

**Figure 5:**
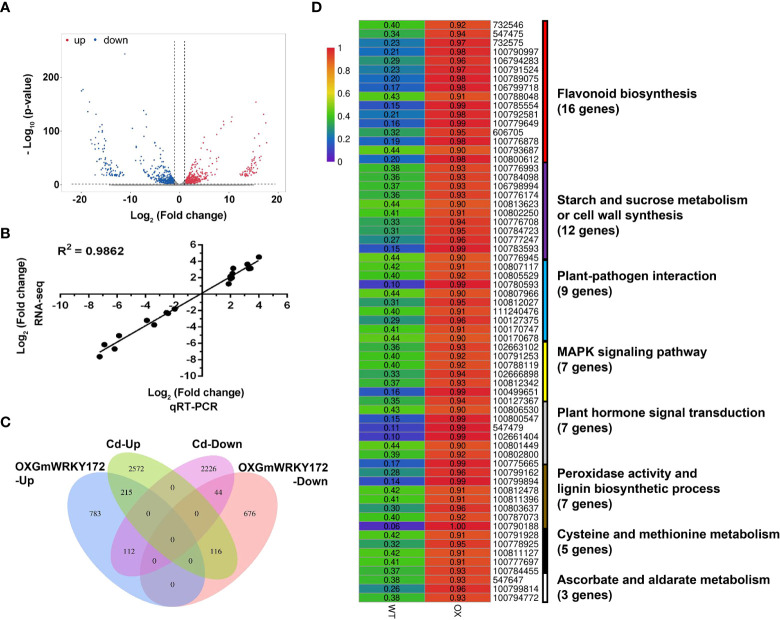
Overexpression of *GmWRKY172* led to extensive transcriptional reprogramming of stress-responsive genes. **(A)** Scatter-plot graphs of the differential gene expression patterns between overexpressing lines and WT. Red represents up-regulated genes, while blue represents down-regulated ones. **(B)** Validation of expression patterns of 20 DEGs by qRT-PCR assay. The correlation is R^2^ = 0.9862. **(C)** Venn diagram shows the overlap of *GmWRKY172* overexpression and Cd-induced DEGs. **(D)** The Cd responsive KEGG pathways among the differentially expressed genes.

To gain deeper insight into the molecular mechanism underlying GmWRKY172-mediated Cd tolerance and Cd translocation, we performed RNA-seq on the *GmWRKY172*-OX and WT plants. In total, 1946 genes displayed differential expression patterns (fold change ≥ 2) in the transgenic lines, including 1110 upregulated genes and 836 downregulated genes (**Figure 5A**, **Table S3**). To validate the RNA-seq results, qRT-PCR analyses were performed to examine the expression of 20 differentially expressed genes (DEGs). As shown in **Figure 5B**, the qRT-PCR results of all tested genes were highly consistent with the RNA-seq data, suggesting that the DEG screening based on RNA-seq is reliable. GO enrichment analysis of the DEGs showed that a total of 141 GO terms (*p* ≤ 0.01) were significantly enriched (**Table S4**, **Figure S4**). Several GO terms associated with Cd tolerance were enriched considerably, such as dioxygenase activity (GO:0051213), oxidation-reduction process (GO:0055114), peroxidase activity (GO:0004601), lignin biosynthetic process (GO:0009809), cell wall organization (GO:0071555), and flavonoid biosynthetic process (GO:0009813) (**Table S4**). Moreover, the DEGs were significantly enriched in 14 different pathways (*p* ≤ 0.05) according to the results of the KEGG pathway analysis. The most enriched pathways are those involved in flavonoid and isoflavonoid biosynthesis, including flavonoid biosynthesis (ko00941), phenylpropanoid biosynthesis (ko00940), isoflavonoid biosynthesis (ko00943), and flavone and flavonol biosynthesis (ko00944) (**Table S5**, **Figure S5**). We then performed a conjoint analysis of the DEGs from this study and the Cd-induced DEGs from our previous study (Cai et al., 2020), and this novel combination revealed that 215 upregulated and 44 downregulated genes were jointly influenced by Cd treatment and *GmWRKY172* overexpression (**Figure 5C**), which are the potential genes targeted by the GmWRKY172-mediated Cd tolerance pathway. KEGG pathway analysis showed that 66 genes were enriched in eight functional terms, which are highly correlated with Cd stress (**Figure 5D**). Of these pathways, the DEGs were similarly and significantly enriched in flavonoid biosynthesis, cell wall synthesis, and peroxidase activity, showing that these three pathways are the primary reasons why *GmWRKY172* overexpression could increase Cd tolerance and decrease Cd translocation.

### 3.5 Overexpression of *GmWRKY172* enhances the antioxidant capacity of soybean and Cd fixation in root cell wall

**Figure 6:**
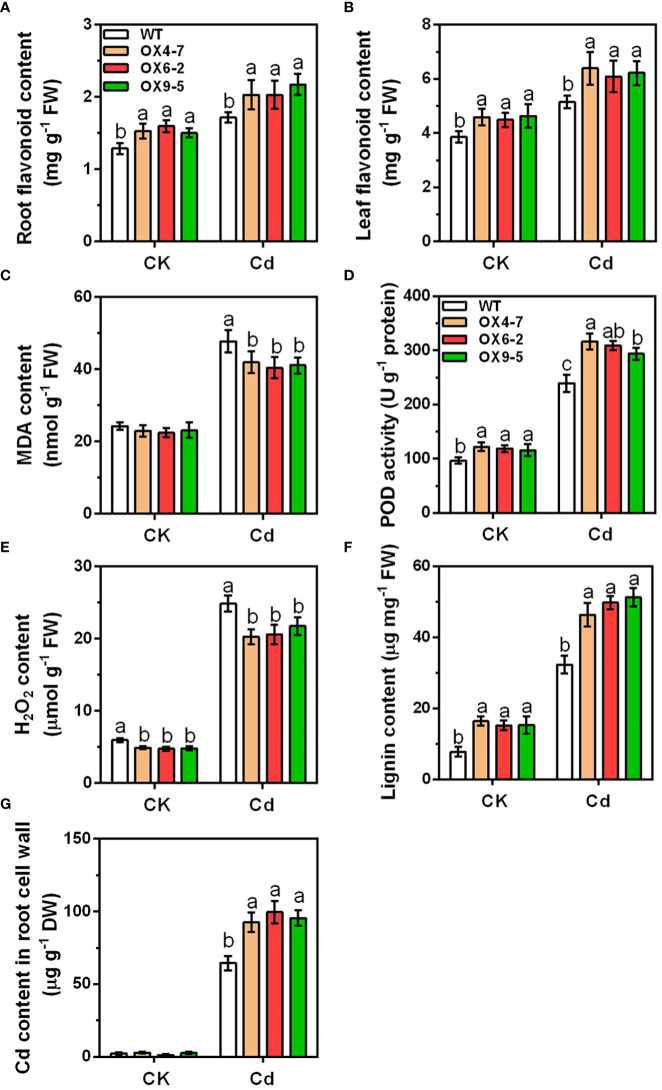
Physiological phenotype analysis of WT and transgenic soybean plants. Root flavonoid content **(A)**, leaf flavonoid content **(B)**, MDA content **(C)**, POD activity **(D)**, H_2_O_2_ content **(E)**, lignin content **(F)**, and the root cell wall Cd content **(G)** were detected under normal or Cd-treated (0.40 mg kg^-1^) conditions. Data are expressed as mean ± SD (*n* = 3). Different letters indicate statistical significance determined by one-way analysis of variance and Duncan’s test (*P* ≤ 0.05).

Since GmWRKY172 directly targets genes involved in flavonoid biosynthesis, cell wall synthesis, and peroxidase activity, we speculated that *GmWRKY172* overexpression may lead to alterations of flavonoid content, antioxidant activity, and cell wall composition. We, therefore, analyzed the related physiological indicators in both WT and transgenic lines to test this hypothesis. As shown in **Figures 6A, B**, the flavonoid contents were significantly increased by Cd stress, and the transgenic lines exhibited significantly higher flavonoid contents than WT. In addition, the transgenic lines also exhibited greater peroxidase (POD) activities than WT, together with less accumulation of malondialdehyde (MDA) and hydrogen peroxide (H_2_O_2_) upon Cd treatment (**Figures 6C–E**). In terms of cell wall synthesis, several genes involved in lignin synthesis were upregulated in transgenic lines (**Figure 5D**). We thus analyzed the lignin contents and found that they were considerably higher in transgenic lines than in WT. The higher lignin content in the transgenic lines may confer stiffness to the root cell wall for Cd resistance (**Figure 6F**). Moreover, the overexpression of *GmWRKY172* in soybean also significantly increased the retention of Cd in the root cell wall when exposed to Cd (**Figure 6F**). These results suggest that GmWRKY172 positively regulates the antioxidant capacity of plants and the Cd fixation in the root cell wall.

## 4 Discussion

### 4.1 Discovery and function of *GmWRKY172*

Transcription factors are universal regulators of all biological processes, including plant development, defense response, aging, rhythm regulation, etc. WRKY TFs are a sizable gene family in the plant genome that work together to form an indispensable regulatory network in the process of controlling numerous stress responses in plants (Rushton et al., 2010). Despite advances in research, our comprehension of the function and mechanism of WRKY TFs in soybean remains incomplete, particularly concerning their involvement in heavy metal stress responses. Using a combination of genome-wide transcriptome analysis and qRT-PCR assay, we previously identified 29 WRKY TFs as being up or downregulated in soybean roots in response to Cd stress (Cai et al., 2020). In this study, we found that the expression of *GmWRKY172* was prominently upregulated by Cd in both roots and leaves (**Figures 1B, C**), suggesting that *GmWRKY172* may play crucial roles in signal transduction under Cd stress.

To further comprehend the role of *GmWRKY172*, overexpressing lines of *Arabidopsis thaliana* and soybean were generated. The overexpression of *GmWRKY172* consistently enhanced the Cd tolerance in both *Arabidopsis thaliana* and soybean (**Figures 2A**, **3A**). The considerable reduction in shoot Cd concentration is a characteristic of the enhanced Cd tolerance in transgenic lines (**Figures 2C**, **3E**), which is similar to other related reports regarding the tolerance of heavy metals (Chen et al., 2019; Tiwari et al., 2020; Zhang et al., 2020a). For example, the expression of PvACR3;1 lowered arsenic accumulations in transgenic rice shoots by 72%-83%, and decreased total arsenic in unhusked rice grain by 28%-39%. Overexpression of the OsHMA3 reduced wheat root-to-shoot Cd translocation by approximately 10-fold and grain Cd accumulation by 96%. Through the analysis of Cd contents in the shoots and roots of plants, we discovered that the overexpressing of *GmWRKY172* could enhance Cd tolerance in plants by decreasing the root-to-seed and root-to-shoot translocation of Cd (**Figures 4G, H**). The possible mechanism could be an increase in the amount of Cd retained in the cell wall or sequestered in the vacuole, which would lead to a reduction in the amount of free Cd available for translocation to the shoot. These two strategies play a role in many plants dealing with Cd stress (Zhou et al., 2020; Li et al., 2021; Zhang et al., 2022). The whole growth period experiments revealed that overexpression of GmWRKY172 in soybean reduces the Cd content in seeds and increases seed size and yield (**Figure 4**). Therefore, it can be concluded that overexpression of *GmWRKY172* enhances Cd tolerance in soybean and reduces the Cd content in seeds by retaining more Cd in roots and limiting its translocation to shoots and seeds.

### 4.2 Molecular mechanisms of *GmWRKY172* in Cd tolerance

RNA-seq has been utilized as a prospective method to investigate the intricate molecular pathways linked with a given phenotype (Shen et al., 2020; Ji et al., 2022; Yan et al., 2022). Global differential gene expression profiling in the overexpressing soybean lines revealed that GmWRKY172 regulates multiple biological pathways. GO and KEGG analyses revealed that the overexpressing line had significantly heightened pathways associated with oxidation-reduction, peroxidase activity, lignin biosynthesis, cell wall organization, and flavonoid biosynthesis (**Figure S4**, **S5**). As the secondary stress of Cd stress, oxidative damage is also extremely detrimental to plant health. The levels of MDA and H_2_O_2_ are commonly employed as indicators to assess oxidative damage. By activating a series of antioxidant enzymes and non-enzymatic antioxidants, plants can maintain cellular redox balance (Kidwai et al., 2019). Besides, cell wall organization and the biosynthetic process of lignin in plants are directly tied to Cd fixation in the cell wall, which is an important strategy for plants to alleviate heavy metal stress by limiting the Cd transport in the cell (Gu and Lan, 2021; Yuan et al., 2022). These results indicated that the overexpression of *GmWRKY172* in soybean results in extensive transcriptional reprogramming of Cd stress-responsive genes, leading to enhanced tolerance to Cd.

To further reveal the direct regulatory pathways of GmWRKY172-mediated Cd tolerance, 259 DEGs were identified using conjoint analysis with Cd-induced DEGs in the transgenic soybean line (**Figure 5C**). Bioinformatics analysis showed that eight functional terms highly correlated with the Cd-stress-responsiveness were enriched within these DEGs (**Figure 5D**), among which flavonoid biosynthesis, cell wall synthesis, and peroxidase activity are consistent with the previous results of GO and KEGG analyses (**Figure S4**, **S5**). Flavonoids are associated with ROS scavenging and can decrease peroxide production, hence enhancing the activity of antioxidant enzymes (Li et al., 2017b). We noticed that overexpressing lines exhibited significantly lower peroxide accumulation and significantly higher flavonoid levels than WT, especially when exposed to Cd (**Figure 6**). It is known that chalcone synthase (CHS) is the enzyme that catalyzes the first step in the flavonoid biosynthesis and ten CHS-encoding genes were found to be significantly upregulated in the transgenic soybean line based on the transcriptome data (**Figure S6**), suggesting that CHS-encoding genes are a class of genes targeted by the GmWRKY172-mediated Cd tolerance pathway. Moreover, it has been revealed that Class III peroxidase (PRX)-encoding genes are implicated in resistance to heavy metals *via* increasing antioxidant activity or the degree of lignification (Wu et al., 2017; Kidwai et al., 2019). As expected, seven PRX genes were also found to be significantly upregulated in the overexpressing lines that had greater levels of antioxidant activity and lignin content (**Figure 6**, **S6**), indicating that the GmWRKY172-PRX cascade module is an important part of GmWRKY172-mediated Cd tolerance.

Here, we have demonstrated that GmWRKY172 overexpression resulted in considerably increased flavonoid, POD, and lignin levels in soybean under Cd treatment. Interestingly, a few CHS and PRX genes involved in flavonoid and lignin biosynthesis and ROS-scavenging were also found to be significantly upregulated in the overexpression lines. Based on the aforementioned findings, we envisage the following mechanisms for function of *GmWRKY172* in response to Cd stress. GmWRKY172 positively regulates the Cd tolerance by targeting CHS and PRX genes, strictly controlling the amount of ROS, and protecting cells from oxidative damage. In addition, GmWRKY172 may exert the function of cell-wall reinforcement and restriction of Cd translocation from roots to shoots by modulating the expression level of PRXs and subsequently increasing lignin content. Although several CHS and PRX genes were upregulated in the overexpression lines, whether these genes are directly regulated by GmWRKY172 is still unclear and needs a more detailed investigation.

### 4.3 Reducing Cd accumulation in edible parts is crucial for controlling Cd intake

The prevalent Cd contamination in soil threatens human health through food chains (Rizwan et al., 2016). When Cd accumulates in the edible parts of plants, such as grains, vegetables, and fruits, it can pose a significant health risk to humans who consume them. Chronic exposure to Cd can cause kidney damage, bone mineral loss, and increase the risk of cancer (Järup and Akesson, 2009). Therefore, it is imperative to reduce Cd accumulation in the edible parts of crops to ensure food safety. One approach is to modify the soil through phytoremediation and the application of amendments such as lime, phosphate, and organic compounds to reduce Cd availability for plant uptake (Bolan and Duraisamy, 2003; Guo et al., 2018; Rizwan et al., 2018). Good agricultural practices including the irrigation management,the strict application of fertilizer, and the limitation of Cd content in fertilizer can also contribute to the reduction of Cd contamination during crop production (Sterckeman et al., 2018; Ulrich, 2019; Mubeen et al., 2023).

Another strategy involves the breeding of Cd-tolerant and low Cd crops. While researchers have explored various approaches to achieve this objective, the studies about the genes that are able to increase Cd tolerance and reduce Cd accumulation simultaneously are far from enough at present. In crops, the Cd contents of shoot and seed depend on the absorption of Cd from roots and its translocation from roots to shoots or to seeds. As a transcription factor, GmWRKY172 can regulate various Cd-induced biological processes (**Figure 5D**) and has been proven to increase the tolerance of Cd in *Arabidopsis* and soybean (**Figures 2**, **3**). More importantly, *GmWRKY172*-overexpressing lines increased the yield of soybean and reduced seed Cd accumulation under Cd stress when compared to the WT (**Figure 4**). Since soybean is a soil-friendly and protein-rich crop (Hua et al., 2020), it is vital to investigate Cd tolerance-related genes and their mechanisms to develop Cd-tolerant and low Cd soybean variety. In summary, reducing Cd accumulation in edible parts of crops is critical to ensure food safety. By implementing a combination of soil management, good agricultural practices, and breeding programs, we can reduce Cd accumulation in crops and ensure a safe and healthy food supply.

## Data availability statement

The datasets presented in this study can be found in online repositories. The names of the repository/repositories and accession number(s) can be found below: https://www.ncbi.nlm.nih.gov/, PRJNA913970.

## Author contributions

PX, YY, CX, ZG, ZH, YZ, and ZC performed the experiments and date analyses. PX, IA, ZC, and HN prepared the manuscript. HN planned, supervised and financed this work, as well as edited the manuscript. All authors contributed to the article and approved the submitted version.
