# Supplementary material for: Overexpression of GmWRKY172 enhances cadmium tolerance in plants and reduces cadmium accumulation in soybean seeds
Source: Front Plant Sci. 2023 Mar 9;14:1133892. doi: 10.3389/fpls.2023.1133892 (PMC10033887; doi:10.3389/fpls.2023.1133892)
Supplement: Supplementary file 1 [file DataSheet_1.pdf]

Supplementary Material

Overexpression of *GmWRKY172* Enhances Cadmium Tolerance in Plants and Reduces Cadmium Accumulation in Soybean Seeds

Peiqi Xian[1,2,3#], Yuan Yang[1,2,3#], Chuwen Xiong[1,2,3#], Zhibin Guo[1,2,3], Intikhab Alam[4], Zihang He[1,2,3], Yakun Zhang[1,2,3], Zhandong Cai[1,2,3,4*], and Hai Nian[1,2,3,5*]

*** Correspondence:** Zhandong Cai (zdcai@scau.edu.cn), Hai Nian (hnian@scau.edu.cn).

1 Supplementary Data

1.1 CDS sequencing information of *GmWRKY172*

ATGGCAAAACTTGGTTGTTCTAGTCGTAGGAAAGCAATTGAGGAGCTCCTTAGAGGGCG
TGATTGTGCCAAACAACTTAGGAGTGTCATCAATGGGAGTTGTGAAGATGGATCATCAA
CAACCCCATCATTTGCTGAACAACTTGTGAAGGAGGTGCTCATGTCCTTCACCAACTCCC
TCTCGTTCTTGAAGAACAACCCCACTTCTGAATCCCATGATGTTTCCAATGTTCAAGTTT
GTGAATCTCCCAAGTCTGAGGACTCCCAAGAGAGCAATTGCAAGAGCTCCATCATTAAG
GAACGTAGAGGGTGCTACAAGAGAAGAAGAACTGAACAAACATGGGAGAAGGAATCTG
AAGCTCCAATTGATGACGGCCATCAGTGGAGAAAGTATGGCCAAAAGGAGATCCTGAG
TGCCAAATTCCCAAGGAACTACTATAGATGCACTCACAAATTTGACCAAGGTTGCCAAG
CAACAAAACAGGTGCAAAGAGTTCAAGAGGAACCAATCCTATACAAGACCACCTACTA
TGGCCTCCACACTTGCAAGAACTTGGCAAACCCTGAGATCATACTTGACCCTATGTCCCC
TTCATCCTCATCCAAGTTCCTTAGCTTTGACAACTCCTTCCCAACCCCATCAAAGCAAGA
GTGCCCCTTTCTCTCATCTTCTAATTTGCCATCATCAGTGAAAGGGGAGTGCAAGGAGGA
GGTCCCTCCCTCAACTTCCTCCAATCATTATCTCATCTCTTCTGACCTCACTTTTGATAGC
TCACCAAGGCATCATGTCACTCTATCATCAACACTTGACTCAGAGTACAAGAGTGTGGA
CATTTCGGATGTCTTGTATGATTCTGCTCAGCTTGATGATGCCTTTGAACCCTTCCTTGAA
TTTAGATGA

2 Supplementary Figures and Tables

2.1 Supplementary Figures

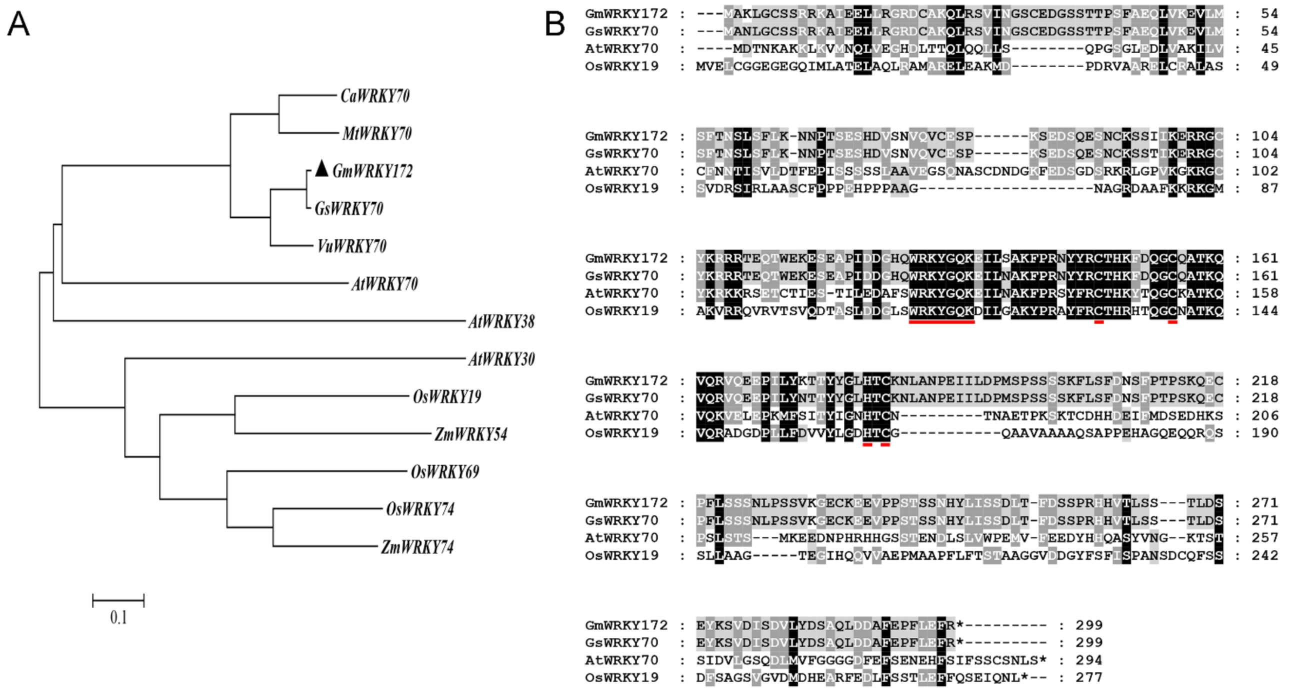

Supplementary Figure S1. Phylogenetic analysis and multiple sequence alignment. (A) Phylogenetic analysis of WRKY proteins in plants. All the available peptide sequences and the gene identifiers of the proteins were obtained from the Phytozome 13 (https://phytozome-next.jgi.doe.gov/). The WRKY proteins including *Cicer arietinum* (CaWRKY70; PAC:40154257), *Medicago truncatula* (MtWRKY70; PAC:31081045), *Glycine max* (GmWRKY172; PAC:41165213), *Glycine soja* (GsWRKY70; PAC:41178155), *Vigna unguiculata* (VuWRKY70; PAC:39022639), *Arabidopsis thaliana* (AtWRKY70; PAC:37412483, AtWRKY30; PAC:37423781, AtWRKY38; PAC:37417844), *Oryza sativa* (OsWRKY19; PAC:33159599, OsWRKY69; PAC:33106462, OsWRKY74; PAC:33142676), and *Zea mays* (ZmWRKY54; PAC:40259137, ZmWRKY74; PAC:40189374). (B) Multiple sequence alignment of WRKY proteins from *Glycine max* (GmWRKY172), *Glycine soja* (GsWRKY70), *Arabidopsis thaliana* (AtWRKY70), and *Oryza sativa* (OsWRKY19). The red underline marks the WRKYGQK motif and C2-HC zinc finger structure.

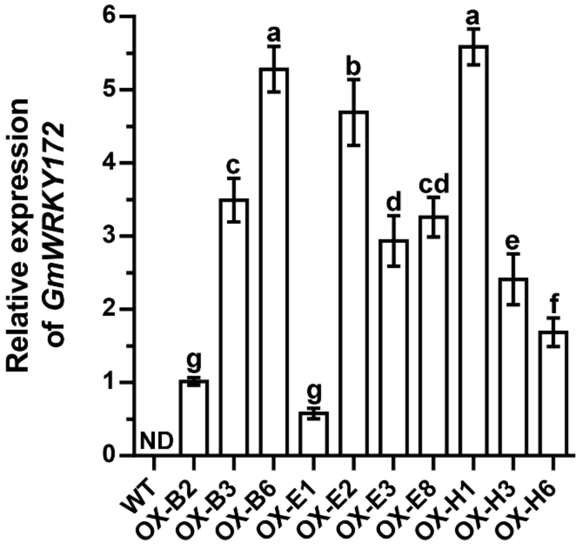

Supplementary Figure S2. Quantitative real-time PCR analysis of overexpressing *GmWRKY172 Arabidopsis* lines. ND, not detected. Data are expressed as mean ± SD (n = 3). Different letters indicate statistical significance determined by one-way analysis of variance and Duncan's test ($P \leq 0.05$).

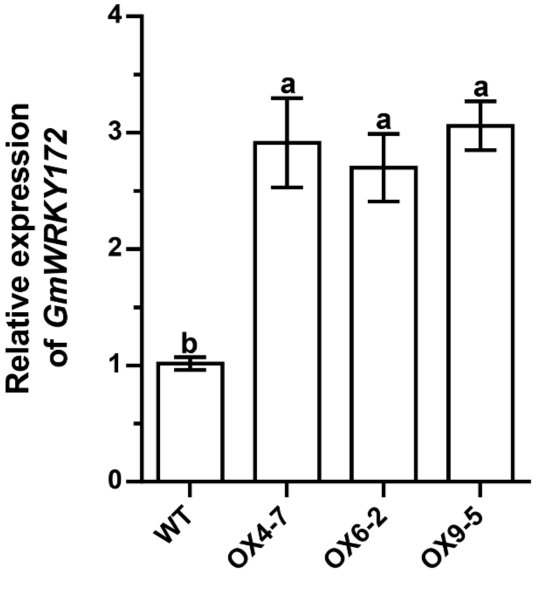

Supplementary Figure S3. Quantitative real-time PCR analysis of overexpressing *GmWRKY172* transgenic soybean lines. Data are expressed as mean ± SD (n = 3). Different letters indicate statistical significance determined by one-way analysis of variance and Duncan's test ($P \leq 0.05$).

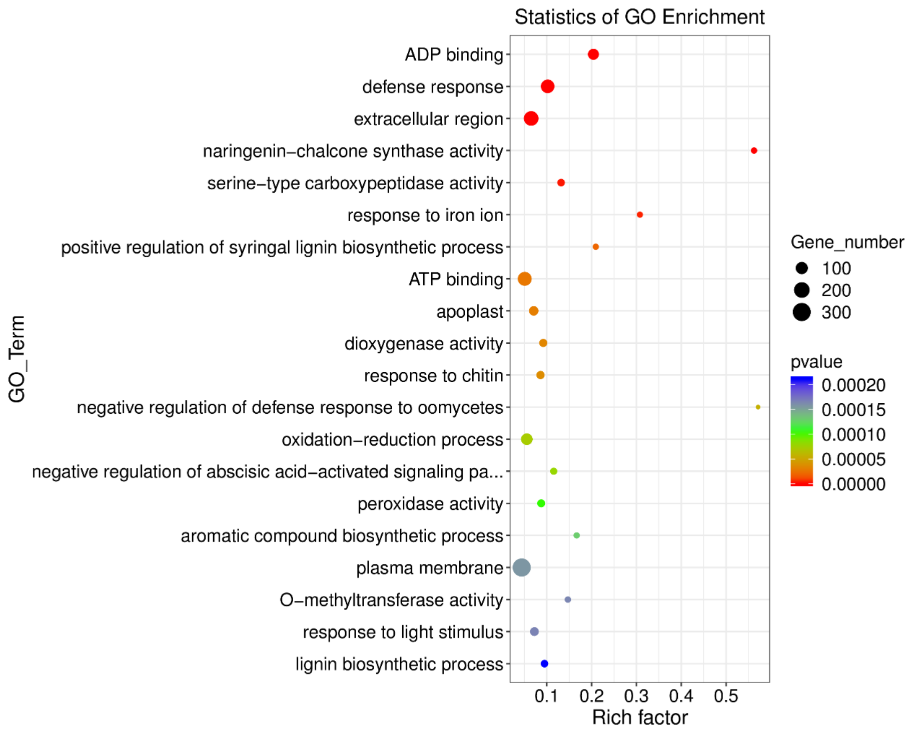

Supplementary Figure S4. GO analysis of the differentially expressed genes (DEGs).

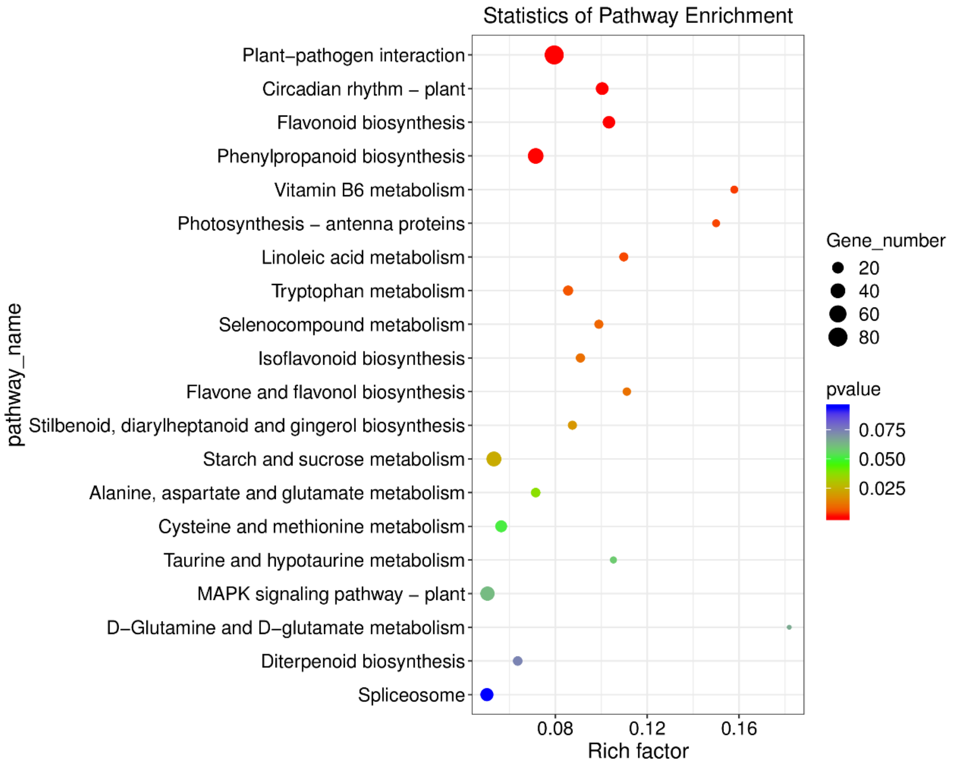

Supplementary Figure S5. KEGG analysis of the differentially expressed genes (DEGs).

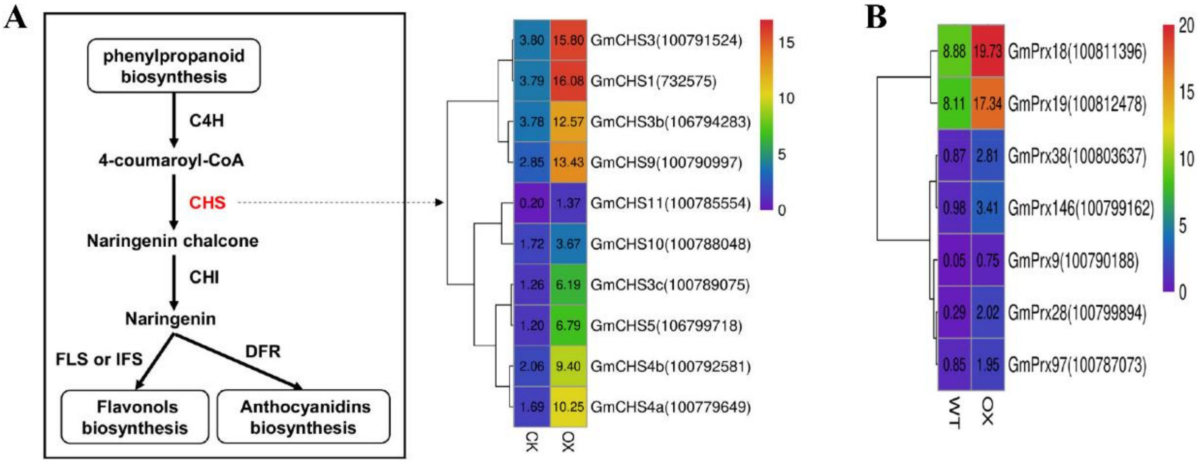

Supplementary Figure S6. Ten chalcone synthase (CHS)-encoding genes (A) and seven Class III peroxidase (PRX)-encoding genes (B) were significantly upregulated in GmWRKY172-overexpressing lines.
